# Short latency afferent inhibition differs with load type during isometric finger abduction

**DOI:** 10.1186/s40101-026-00424-y

**Published:** 2026-02-25

**Authors:** Kangjing Yang, Tatsunori Watanabe, Takayuki Horinouchi, Sumi Miyoshi, Jingnan Li, Kazuya Saita, Shota Date, Hikari Kirimoto

**Affiliations:** 1 Department of Sensorimotor Neuroscience, Graduate School of Biomedical and Health Sciences, Hiroshima University, 1-2-3 Kasumi, Minami-Ku, Hiroshima, 734-8553 Japan; 2 Graduate School of Health Sciences, Aomori University of Health and Welfare, 581 Mase, Hamadate, Aomori 030-8505 Japan; 3 Japan Society for the Promotion of Science, Tokyo, Japan; 4 Department of Psychosocial Rehabilitation, Graduate School of Biomedical and Health Sciences, Hiroshima University, Hiroshima, Japan; 5 Department of Analysis and Control of Upper Extremity Function, Graduate School of Biomedical and Health Sciences, Hiroshima University, Hiroshima, Japan; 6 Computational Behavioral Neuroscience Laboratory, Nara Institute of Science and Technology, Nara, Japan

**Keywords:** Short-latency afferent inhibition, Heteronymous short latency reflex, Heteronymous long latency reflex, Primary motor cortex, Sensorimotor integration

## Abstract

**Background:**

Static muscle contraction involves two distinct load types. One type, called a position task, entails holding the limb in a fixed position while counteracting an inertial load, while the other type, known as a force task, involves exerting a consistent force against a solid constraint. While proprioceptive information has been shown to be required more during the position task, it has remained to be elucidated how sensorimotor integration differs between these two tasks.

**Methods:**

This study investigated differences in short latency afferent inhibition (SAI) and heteronymous reflex responses between the force and position conditions. Sixteen participants performed static contractions of the first dorsal interosseous (FDI) muscle. In the force task, they exerted a constant force corresponding to 10% maximum voluntary contraction (MVC) against a rigid restraint. In the position task, they sustained a target abduction angle of 20° while holding a load equivalent to 10% MVC. SAI was induced by the paired application of electrical stimulation to the right median nerve and transcranial magnetic stimulation over the left motor cortex at an N20 + 2 ms interval. Motor evoked potentials (MEPs) were recorded from the FDI muscle to quantify the magnitude of SAI. Heteronymous short and long latency reflexes (SLR and LLR) were also examined, and their amplitudes were compared between the force and position tasks.

**Results:**

SAI was significantly attenuated in the position task (*p* < 0.05). Additionally, SLR and LLR amplitudes were significantly greater during position task (*p* < 0.05).

**Conclusions:**

These findings suggest distinct sensorimotor processing strategies depending on the load type.

**Supplementary Information:**

The online version contains supplementary material available at 10.1186/s40101-026-00424-y.

## Introduction

Exerting a constant force by resisting a rigid constraint (force task) and maintaining a steady limb angle under an identical inertial load (position task) involve distinct neural control strategies, despite producing comparable net muscle torque according to Newtonian mechanics. For example, previous studies [1–3] assessing the first dorsal interosseous (FDI) muscle reported greater short-latency reflex (SLR) amplitude during the position than force task following median nerve stimulation. Because SLR amplitude reflects presynaptic inhibition of Ia afferents [4, 5], this finding suggests enhanced heteronymous afferent input to the FDI motor neuron pool due to reduced presynaptic inhibition during the position task. Furthermore, the long-latency reflex (LLR) amplitude has been demonstrated to be greater in the position than force task following median nerve stimulation. Although the LLR descends via the corticospinal tract, after ascending through the thalamocortical pathway, and is regulated by the basal nuclei [6, 7], recent study suggested that task-related differences in LLR amplitude should not be directly interpreted as evidence of differences in sensory information processing within the central nervous system or in the primary motor cortex (M1) excitability [8]. Meanwhile, somatosensory evoked potential (SEP) gating, indicated by a reduction in SEP amplitude, was greater during the position than force task following ulnar, but not median, nerve stimulation [2], suggesting that proprioceptive processing depends on the load type during static muscle contraction. Nevertheless, how different load types influence sensorimotor integration—the process by which the central nervous system links sensory input to motor output—remains unclear.

A commonly utilized method for evaluating sensorimotor integration is short latency afferent inhibition (SAI), a protocol based on transcranial magnetic stimulation (TMS) [9–11]. SAI is characterized by suppression in motor evoked potential (MEP) amplitude following conditioning afferent electrical stimulation delivered to a peripheral mixed nerve [10, 11]. Specifically, when the interval between the peripheral conditioning stimulus and the TMS pulse applied to the M1 slightly exceeds the N20 latency of SEPs, the conditioned MEP amplitude is attenuated relative to its unconditioned response [9]. This reduction is mediated by cholinergic neurons that facilitate GABAergic interneuron activation, leading to suppression of pyramidal neurons during sensory information transfer from the primary somatosensory cortex (S1) to M1 [12–14]. The magnitude of SAI has been reported to vary significantly depending on task conditions [15–17].

Accordingly, the present study aims to investigate whether the modulation of SAI differs between the force and position tasks. Given that the position task requires more proprioceptive information than the force task, we hypothesized that the ascending sensory input generated by peripheral nerve electrical stimulation would be more strongly inhibited during the position task, resulting in attenuated SAI. From a physiological anthropology perspective, task-specific modulation of SAI may provide valuable insight into the fundamental mechanisms by which humans adapt to different load conditions through sensory processing adjustments.

## Materials and Methods

### Participants

A priori power analysis was conducted using G*Power (version 3.1.9.7) [18] to determine the sample size required to detect differences in the primary outcome (SAI inhibition (%)). Based on a Cohen’s f = 0.4 (representing a medium-to-large effect size based on prior literature) [2, 19, 20], an α of 0.05, and a power of 0.8, a minimum of 12 participants was required. To ensure robust results and account for potential data loss, we recruited 16 participants through a notification on our laboratory homepage. Sixteen healthy students from Hiroshima University (12 males and 4 females, 24.12 ± 3.85 years old) participated in this study between January 6 and March 31, 2024. All participants were confirmed to be free of diseases and dysfunctions of the nervous and motor systems. The handedness of participants were assessed using the Oldfield Inventory scores (score range: 0.9–1.0), confirming that all participants were right-handed [21]. Additionally, all individuals demonstrated either normal or corrected-to-normal vision. Informed written consent was secured from each participant prior to initiating the experimental procedures. Ethical approval was granted by the Ethics Committee of Hiroshima University (No. E-2261) and the study adhered to the principles outlined in the Declaration of Helsinki.

### Experimental setup and tasks

**Fig. 1:**
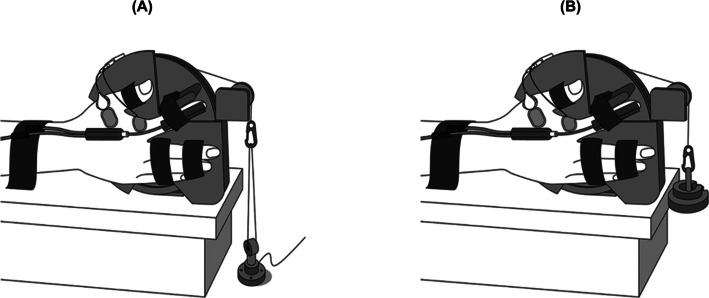
Illustration of the experimental setup depicting the force and position tasks. The apparatus involved a rotating wheel linked to either a force transducer (**A**) or inertial load (**B**) through a pulley system and nylon cord. An electro-goniometer was attached to the right hand during the position task to measure the angle of metacarpophalangeal joint abduction [20]

Participants were seated upright with their right hand positioned in the custom-designed device that has been used in our prior research methodologies (Fig. 1) [2, 20]. Both the force task and the position task were performed with the index metacarpophalangeal joint maintained at 20° of abduction. The apparatus included a rotating wheel linked to either a force transducer (TU-QR, TEAC, Tokyo, Japan) or an inertial load connected via a pulley and a nylon line. The participant’s posture was carefully standardized: the right shoulder was abducted at 10–20°, the elbow was maintained at a flexion angle of 110°, and the forearm and wrist were stabilized in a neutral alignment to minimize compensatory movements. The right index finger was secured to a bar attached to the wheel (7.5 cm diameter), aligning the rotational axis of the wheel with the metacarpophalangeal joint’s rotational axis for precise movements. Flexion and extension of the metacarpophalangeal joint and interphalangeal joints were constrained, allowing only abduction–adduction. In addition, the thumb was abducted to an angle of 45°, and the remaining fingers were fixed at fully extended positions. We carefully observed that this posture was always maintained while the experiment was being conducted.

The participants engaged in two submaximal contraction tasks at equivalent torque levels. During the force task (Fig. 1A), they sustained a force level of 10% of their maximum voluntary contraction (MVC). During the position task (Fig. 1B), they resisted an inertial load corresponding to 10% MVC while maintaining the target 20° joint position. An electro-goniometer (SG65, Biometrics, Gwent, UK) measured the abduction–adduction angle during the position task. Visual feedback related to joint angle and force was displayed on a monitor (LCD-MF235XDBR, I–O Data, Japan) located 1 m ahead of the participants, and the online visual feedback was shown as red line for the force task and blue line for the position task progressing with time from left to right using LabChart 8 (AD Instruments, Bella Vista, Australia). The participants were instructed to match their force or position to a gray horizontal target line and maintain the target force or angle as steadily and accurately as possible. The feedback gain was calibrated to 2.5%/cm, corresponding to the MVC level for the force tasks and full range of motion about the metacarpophalangeal joint for the position tasks [2, 20, 22, 23]. The force and electro-goniometer signals were low-pass filtered at 20 Hz and digitized at 10 kHz (PowerLab, AD Instruments, Bella Vista, Australia).

### N20 latency measurement

An Ag/AgCl electrode was positioned 2 cm posterior to the C3 (C3’) site according to the international 10–20 system, with a reference electrode attached to the right earlobe. A ground electrode was placed on the right wrist, slightly proximal to the stimulation electrode, to ensure high-quality recordings. The right median nerve was stimulated using a bipolar electrode, with the anode placed distally. A total of 300 stimulations were delivered using a constant current stimulator (Digitimer DS3, Digitimer, Welwyn Garden City, UK) as 0.2-ms square-wave pulses at 3.3 Hz. Stimulus intensity was set at 1.2 times the motor threshold required to evoke a contraction of the right FDI muscle. The evoked potentials were amplified (FA-DL-160, 4 Assist, Tokyo, Japan), band-pass filtered (1.6–500 Hz), and digitized at a sampling rate of 10 kHz (PowerLab). Recorded signals were averaged across 300 epochs to calculate the latency of SEP N20 component.

### MEPs and SAI recordings

EMG signals were acquired using single-use disposable pre-gelled Ag/AgCl surface electrodes placed over the muscle belly of the FDI to maintain a high signal-to-noise ratio and ensure consistent recording quality. These EMG signals were amplified (× 100; FA-DL-160, 4 Assist, Tokyo, Japan), band-pass filtered between 5 and 500 Hz, and digitized at 10 kHz (PowerLab). The data were stored for off-line analysis (LabChart 8, AD Instruments, Bella Vista, Australia).

Three conditions were tested: rest, force task, and position task. TMS was delivered using a 70-mm figure-of-eight coil connected to a monophasic magnetic stimulator (Magstim 200, Magstim, Carmarthenshire, UK). The coil was aligned over the left M1 and maintained at 45° to the sagittal plane. The motor hotspot was located as the position where suprathreshold stimulation consistently elicited the largest MEPs in the FDI muscle. Test stimulus (TS) intensities were defined as the minimal stimulation intensities required to elicit MEPs of approximately 1 mV at rest for the rest condition and during voluntary contraction for the force and position tasks. These intensities were determined using an incremental and decremental method in steps of 1–2% of the maximum stimulator output (MSO), until the 1 mV MEPs were consistently observed in at least 5 out of 10 consecutive trials.

SAI was elicited by brief suprathreshold electrical stimulation of the right median nerve (0.2-ms square-wave pulse) delivered via a constant-current stimulator (Digitimer DS3) prior to TMS. Stimulus intensity was set at 1.2 times the motor threshold required to evoke a visible contraction in the right FDI muscle. The interstimulus interval between peripheral electrical stimulation and TMS was set to the latency of the N20 + 2 ms, and the intertrial interval was 4000 ± 200 ms. Two blocks were conducted for each condition (rest, force task, and position task). Each block consisted of 12 TMS trials, with median nerve stimulation applied randomly in 6 trials. Thus, for each condition, 12 conditioned and 12 unconditioned MEPs were obtained. Conditions were performed in a randomized order, and blocks were separated by a rest period of 60 s to avoid fatigue.

### SLRs and LLRs

Electrode position and stimulus intensity for right median nerve stimulation were the same as described above. This approach enables evaluation of the agonist response to feedback from low-threshold afferent inputs without activating the antagonist muscle, minimizing contamination from F-wave [24]. Stimulation frequency and duration were set to 1.3 Hz and 1 ms, respectively. Reflexes were recorded 100 times in two blocks of 50 stimulations each, for both the position and force tasks in a randomized order. Blocks were separated by 60-s rest periods to avoid fatigue. Surface EMG signals were recorded from the FDI as described above, with the band-pass filter set to 1–3000 Hz.

### Protocol

At the beginning of the session, we assessed MVC of the right index finger abduction. The procedure for assessing MVC followed the previously established protocol [2, 20], where participants gradually increased their force output from rest to maximum over 3 s and held the peak force for an additional 3 s with verbal encouragement. MVC was measured at least three times for each participant with 90-s rest between trials. If the peak of force values across trials exhibited a variation within 5%, the highest value was considered as the MVC. Otherwise, additional trials were conducted until a variation of 5% or less was achieved (the MVC trials were carried out 3–5 times).

**Fig. 2:**
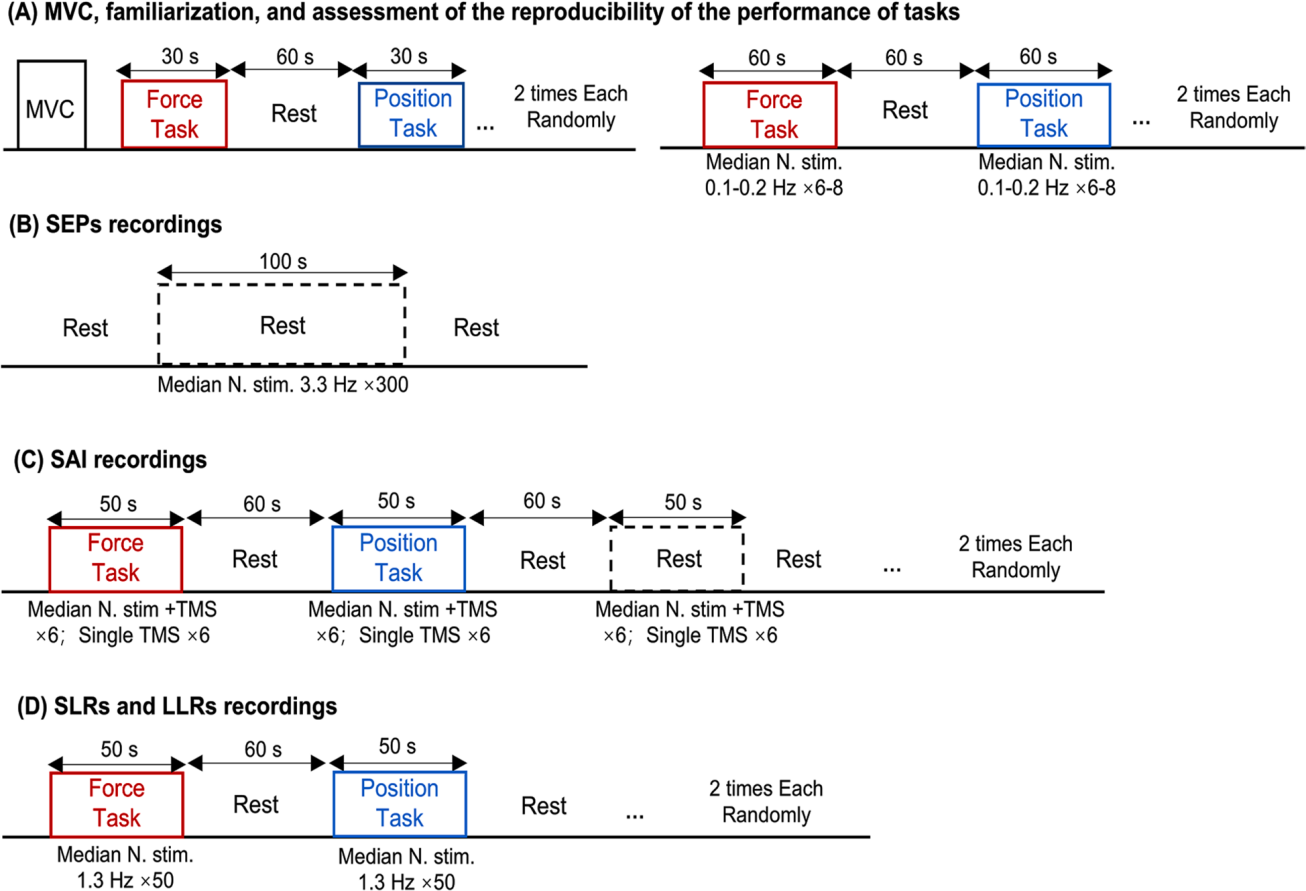
Experimental procedure. Experimental procedure for MVC and familiarization of tasks (**A**), the N20 components of SEPs measurement (**B**), SAI recordings (**C**), and heteronymous reflex responses recordings (**D**). During SAI measurement, TMS was presented in a random order with and without conditioning electrical stimulation to the median nerve. The recordings of SAI and SLRs/LLRs were conducted in separate submaximal contraction trials in a randomized order

Next, to familiarize the participants with the tasks, they performed 30-s static contractions of the FDI muscle at 10% MVC for the force task and at 20°abduction for the position task in a randomized order, with a 1-min rest between trials. Task accuracy was monitored by assessing the absolute error of torque in the force task and joint angle in the position task. If the error exceeded 5%, participants repeated the practice until performance fell within the predefined range (2–5 trials). Participants then performed 1-min static contractions for each task twice in a randomized order, separated by a 1-min rest periods, during which median nerve stimulation was applied at 0.1–0.2 Hz with 6–8 stimulations per task (Fig. 2A). These contractions were used to reassess absolute error and EMG amplitude during the tasks.

After the familiarization and reassessment, the latency of the N20 component of SEPs was measured at rest (Fig. 2B). SAI was then recorded at rest and during both tasks (Fig. 2C), whereas the SLRs and LLRs were recorded during both tasks (Fig. 2D). SAI and SLR/LLR recordings were conducted in separate submaximal contraction trials in a randomized order. To minimize the influence of transient force fluctuations, stimuli were delivered when the force and position signals had reached their respective targets and remained stable for at least 1 s. Throughout the recordings, EMG activity of the right FDI muscle was monitored to ensure consistency between tasks.

### Data analysis

To quantify the maximum EMG activity of the right FDI muscle, EMG activity during MVC trials was rectified and averaged over a 1-s window centered on the peak force. The EMG recorded during static contraction in two tasks was normalized to the EMG amplitude during MVC (%EMG).

Using data during task familiarization, we evaluated the absolute error rate (AER) and background EMG (BEMG) during the two tasks. Specifically, we calculated the average EMG amplitude and force/angle values during the middle 20 s of a 30-s static contractions, without median nerve stimulation, at 10% MVC in the force task or at 20° of abduction in the position task. Also, we calculated the average EMG and force/angle values over 1-s period, with 500 ms before and after median nerve stimulation. The AER of force and angle was calculated using the following formula:

$$AER=\frac{\left|Measured Value-Target Value\right|}{Target Value} \times 100$$

Reproducibility of BEMG and AER for the two tasks was verified in all 16 participants. Due to the limited number of channels on the A/D converter and constraints of the analysis software, force and angle were not recorded during the SAI and SLR/LLR recordings. For the SAI and SLR/LLR measurements, only the reproducibility of BEMG was evaluated.

The magnitude of SAI was quantified as the percentage of inhibition, calculated as follows:

$$SAI inhibition (\%) =\frac{\left|Unconditioned MEP - Condition MEP\right|}{Unconditioned MEP} \times 100,$$

where 0% indicates no change (unconditioned equals conditioned MEPs).

To evaluate the BEMG during SAI recording, the average amplitude was calculated over a 100-ms window preceding median nerve stimulation.

The amplitudes of SLRs and LLRs were determined by subtracting the average BEMG activity, computed over a 50-ms period preceding right median nerve stimulation, from the peak amplitude after rectifying the EMG [1–3]. They were defined as the difference between baseline and peak values:

$$SLR/LLR amplitude = Peak- BEMG$$

The SLR (20–50 ms post-stimulation) and LLR (50–100 ms post-stimulation) were analyzed over a time window extending from 50 ms before to 200 ms after stimulation. After the automatic removal of trials with excessive artifacts by the software, a total of 100 trails were summed on average to obtain a waveform for each participant and each task.

Statistical analyses were conducted using MATLAB R2023b (The MathWorks, Inc., Natick, MA, USA) and OriginPro 2025 (OriginLab Corporation, Northampton, MA, USA). Results are presented as mean values with standard errors of the mean. We utilized the Shapiro–Wilk test to verify the normality of the data distribution. BEMG amplitudes and the AER with and without median nerve stimulation during the two tasks were compared using two-way analysis of variance (ANOVA). Intraclass correlation coefficients, ICC (2,1), were used to measure the reproducibility of inter-load type BEMG activity for the FDI and the AER of force and angle.

To ensure comparability across conditions during SAI assessment, unconditioned MEP amplitudes were compared among the three tasks (rest, force, and position) using a non-parametric Friedman test. Subsequently, the SAI inhibition (%) was compared between conditions using one-way repeated measures ANOVA. Post-hoc tests were performed using Tukey test for all relevant pairwise comparisons. SLR and LLR amplitudes were compared between the force and position tasks using paired t-test or Wilcoxon signed-rank test, contingent upon the normality of the distribution. To investigate the potential physiological relationship between the modulation of SAI and heteronymous reflexes, Pearson or Spearman correlation were performed among the SAI inhibition (%), SLR and LLR amplitudes. These analyses were conducted both within each task condition and on the task-induced changes (force—position). The significance level was set at *p* < 0.05.

## Results

**Fig. 3:**
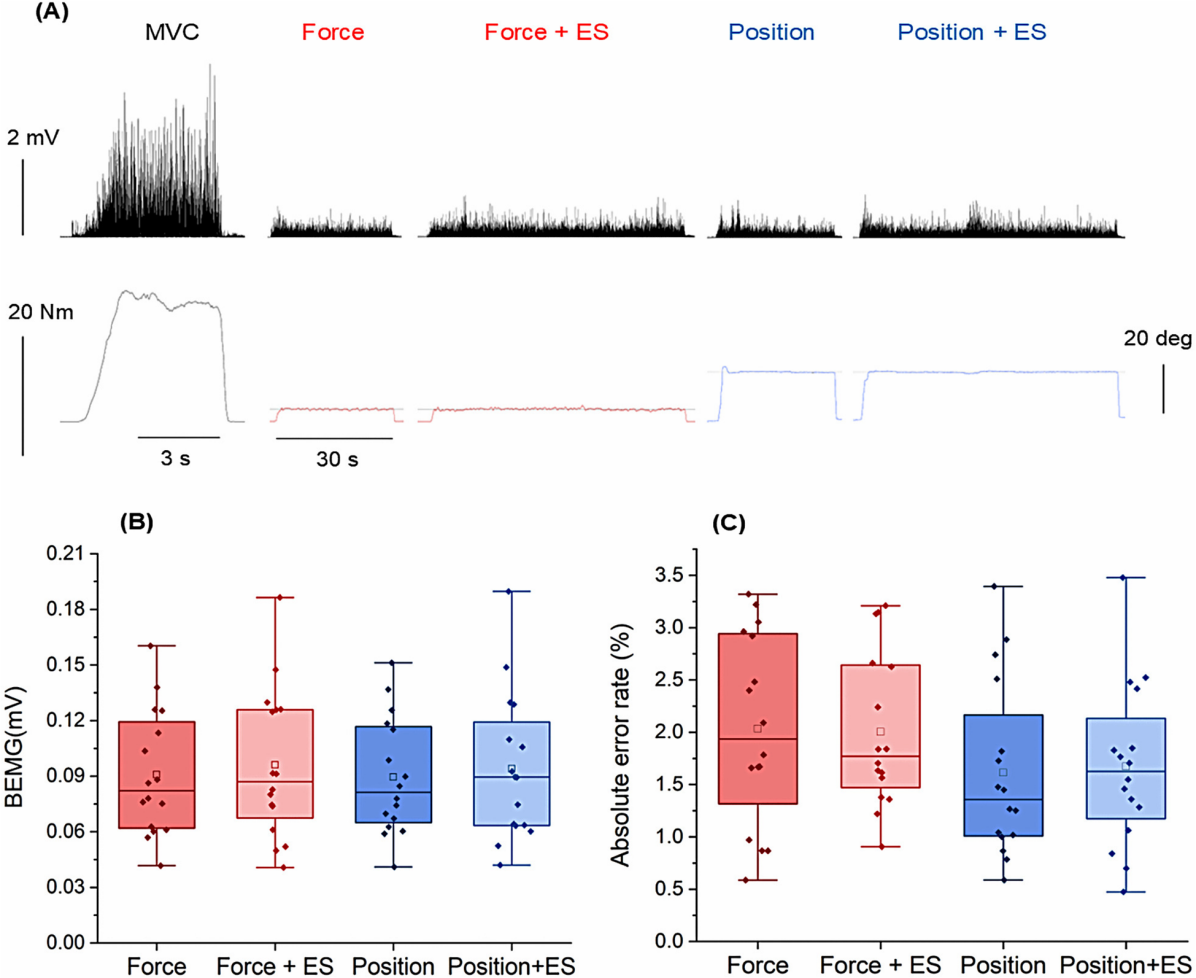
Raw waveforms of EMG, torque, and metacarpophalangeal joint angle during MVC and submaximal static contraction. **A** Raw waveforms recorded from a representative subject during MVC and submaximal static contraction in force and position tasks, with and without median nerve stimulation (ES). EMG activity and muscle torque during MVC are shown in black, muscle torque during the force tasks, with and without ES, is shown in red, and joint angle during the position tasks is shown in blue. **B** Box plots show reproducibility of BEMG. **C** Box plots show absolute error rates (AER) during two tasks, with and without ES

Figure 3A shows raw waveforms of EMG activity, muscle torque, and metacarpophalangeal joint angle recorded from a representative subject during MVC and submaximal static contraction for the force and position tasks, both with and without median nerve stimulation during task familiarization. The mean torque and EMG activity during MVC across all subjects were 0.97 ± 0.08 Nm and 0.90 ± 0.09 mV, respectively. The BEMG of the FDI muscle with and without median nerve stimulation during the force task (0.10 ± 0.01 mV and 0.09 ± 0.01 mV, respectively) and the position task (0.09 ± 0.01 mV and 0.09 ± 0.01 mV, respectively) did not differ significantly (all* p* > 0.076) (Fig. 3B). These EMG activities corresponded to about 11% MVC (force: 11.1 ± 1.2% MVC; position: 11.0 ± 1.1% MVC, *p* = 0.375, Wilcoxon signed-rank test), which was consistent with the load intensity of 10% of maximum muscle strength. Similarly, the AERs with and without median nerve stimulation during the force task (2.0 ± 0.2% and 2.0 ± 0.2%, respectively) and the position task (1.7 ± 0.2% and 1.6 ± 0.2%, respectively) did not show significant differences (all* p* > 0.186) (Fig. 3C).

**Fig. 4:**
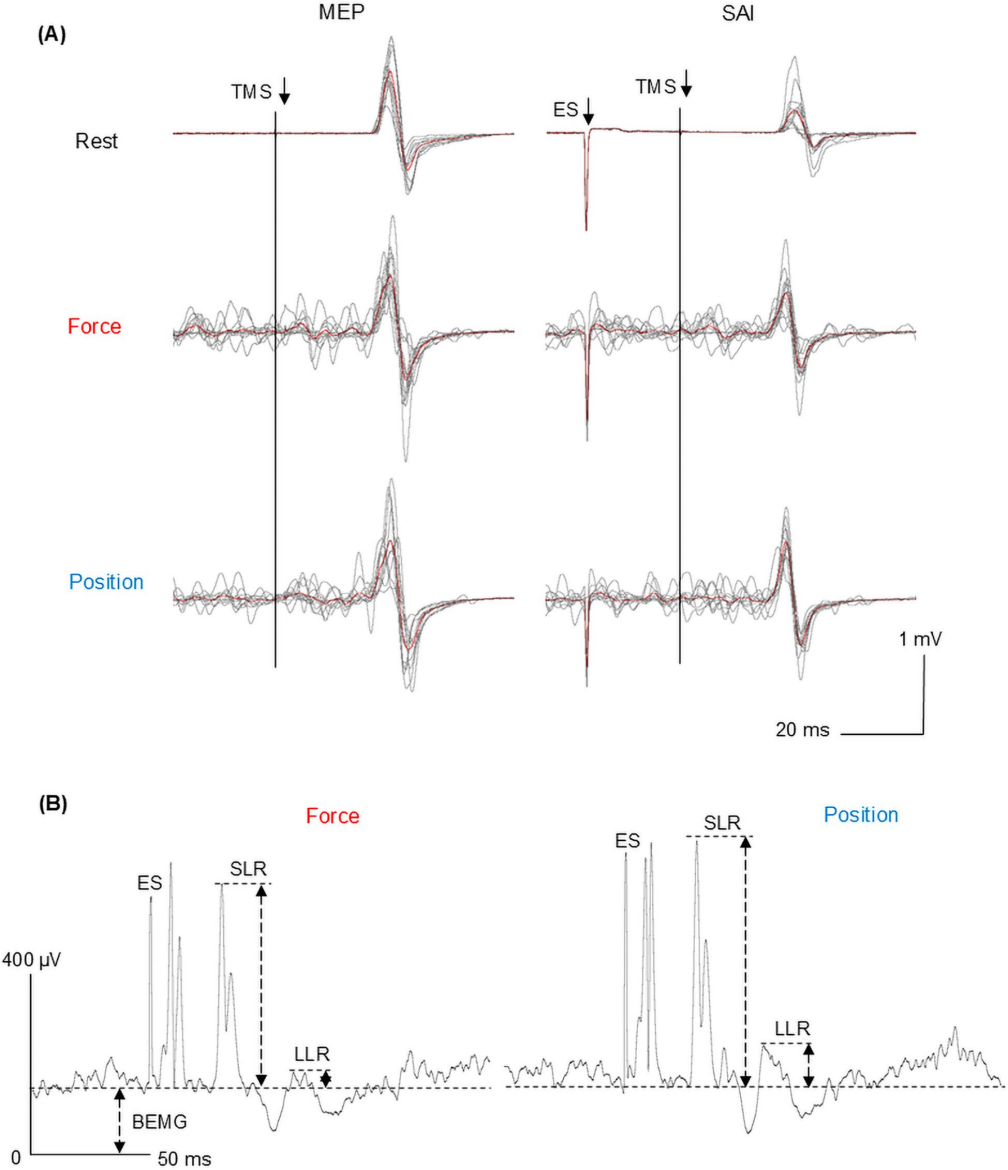
Raw waveforms of SAI and SLRs and LLRs recorded from a representative participant. **A** Representative raw MEP waveforms elicited by single-pulse TMS and TMS with a conditioning electrical stimulation (ES) during rest and the force and position tasks. Light gray lines indicate MEP waveforms from all 12 trials, while thick lines indicate the average waveforms. A reduction in MEP amplitude with conditioned median nerve stimulation (SAI) compared to single pulse MEPs (SAI inhibition (%)) is observed during the position task compared to rest and the force task. **B** Raw, rectified, and averaged EMG waveforms from SLR and LLR recordings during the force and position tasks. The amplitudes of the SLR and LLR are greater during the position task compared to the force task

The mean latency of SEPs N20 component was 19.8 ± 0.5 ms, a representative waveform is provided in Supplementary Figure S1. Figure 4A shows raw MEP waveforms recorded during SAI measurements, both with and without conditioning electrical stimulation at rest and during the force and position task, from a representative participant. Light gray lines indicate MEP waveforms from all 12 trials, while thick lines indicate the average waveforms. Figure 4B shows representative raw, rectified, and averaged EMG waveforms from SLR and LLR recordings during the force and position tasks.

**Fig. 5:**
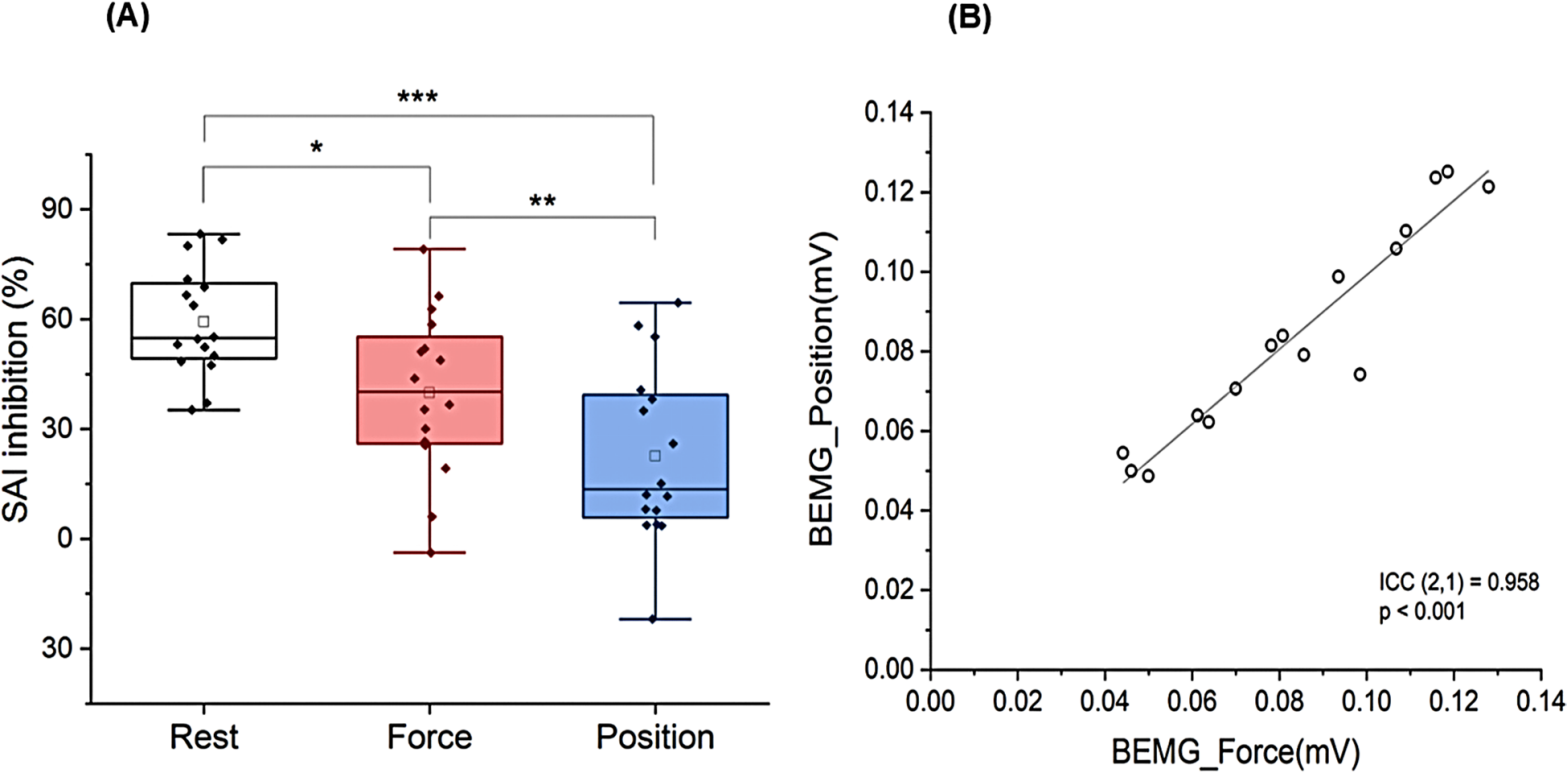
Differences in SAI inhibition (%) between tasks and reproducibility of BEMG. **A** Box plots of SAI inhibition (%) during rest, force, and position tasks. Significant differences are marked with a single asterisk (*p* < 0.05), a double asterisk (*p* < 0.01) and triple asterisk (*p* < 0.001). **B** Intraclass correlation coefficients (ICC) of BEMG between the two tasks

The TS intensities were 55 ± 2%MSO for the rest, 39 ± 2%MSO for the force and position tasks. Conditioning electrical stimulation was delivered at 5.6 ± 0.3 mA. These parameters elicited stable unconditioned MEPs of 1.10 ± 0.08 mV, 1.32 ± 0.11 mV and 1.23 ± 0.12 mV, respectively, with no significant differences observed across conditions (χ^2^_(2)_ = 4.875, *p* = 0.087). As shown in Fig. 5A, the average SAI inhibition (%) was highest during the rest (59.3 ± 3.7%), followed by the force task (39.9 ± 5.6%), and was lowest during the position task (22.6 ± 6.0%). One-way repeated measures ANOVA revealed a significant main effect of task condition on SAI inhibition (%) (F _(2,30)_ = 17.09, *p* < 0.0001, partial η^2^ = 0.533). Post-hoc analysis identified significant SAI inhibition (%) differences among task conditions, with the rest differing from both the position (*p* = 0.0002) and force (*p* = 0.040) tasks, and the force differing from the position tasks (*p* = 0.006). BEMG during SAI recording were 0.08 ± 0.01 mV in the force task and 0.09 ± 0.01 mV in the position task. There was no significant difference between the two tasks (*p* = 0.906), and BEMG was highly reproducible (ICC (2,1) = 0.958, *p* < 0.001).

**Fig. 6:**
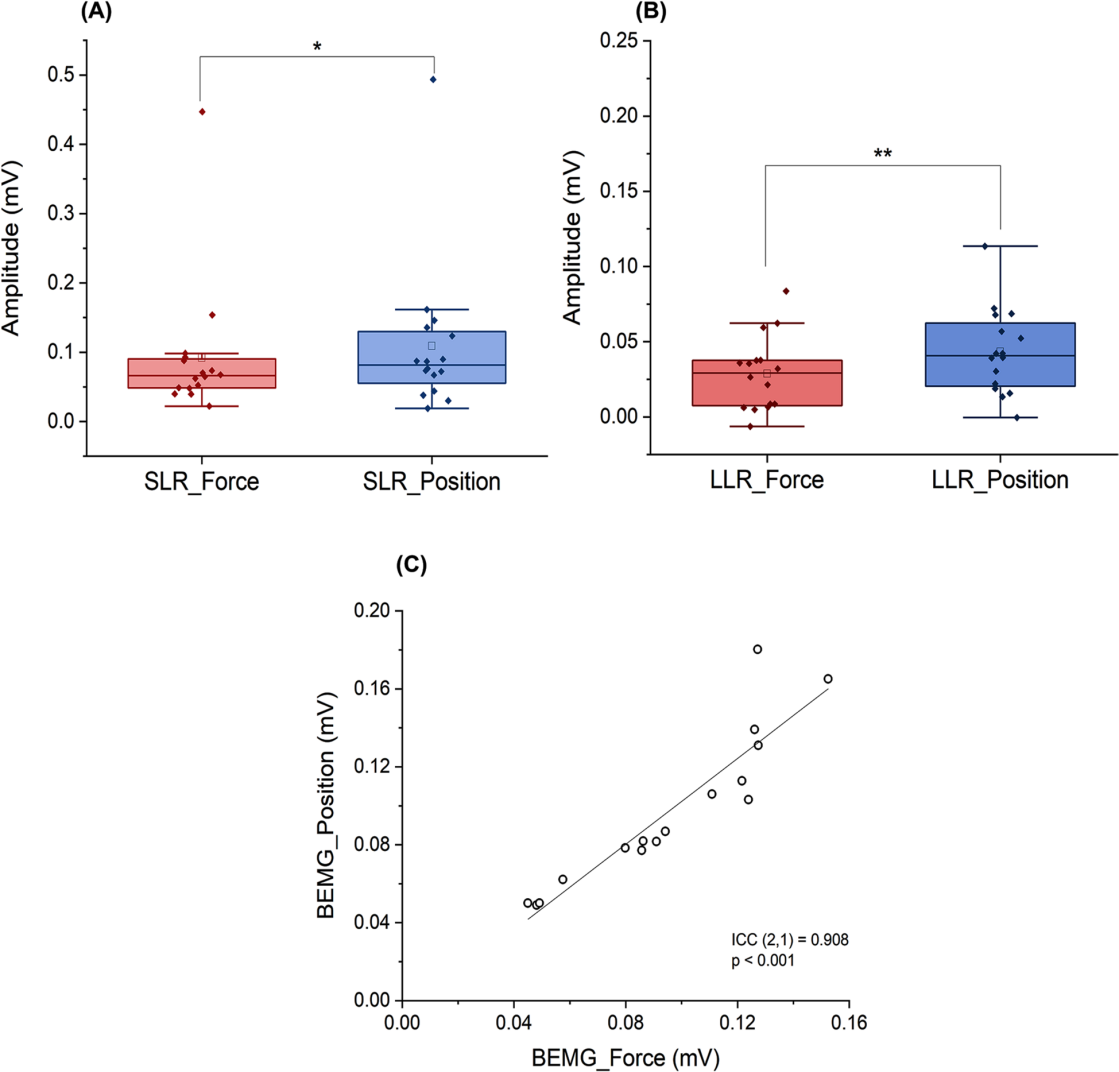
Differences in the amplitudes of the SLRs and LLRs between tasks and reproducibility of BEMG. **A** Box plots comparing reflex amplitudes for SLR between tasks. **B** Box plots comparing reflex amplitudes for LLR between tasks. Statistically significant differences are marked with an asterisk (*p* < 0.05) and a double asterisk (*p* < 0.01). **C** Intraclass correlation coefficients (ICC) of BEMG between the two tasks

The amplitude of SLR was higher during the position than force task (force task: 0.09 ± 0.02 mV, position task: 0.11 ± 0.03 mV; *p* = 0.024, Wilcoxon signed-rank test) (Fig. 6A). The amplitude of LLR was also higher during the position than force task (force task: 0.03 ± 0.01 mV, position task: 0.04 ± 0.01 mV; *p* = 0.003, paired t-test) (Fig. 6B). BEMG during SLR and LLR recordings were 0.10 ± 0.01 mV in the force task and 0.10 ± 0.01 mV in the position task. There was no significant difference between the two tasks (*p* = 0.662), and BEMG was highly reproducible (ICC (2,1) = 0.908, *p* < 0.001).

Correlation analyses revealed no significant associations among SAI and reflex measures across all tested dimensions. In the force tasks, SAI inhibition (%) was not significantly correlated with SLR (*r*_*s*_ = −0.282, *p* = 0.289) or LLR amplitudes (*r* = 0.290, *p* = 0.277). And no significant correlation was found between SLR and LLR amplitudes (*r*_*s*_ = −0.041, *p* = 0.879). In the position tasks, SAI inhibition (%) demonstrated no significant associations with SLRs (*r*_*s*_ = 0.041, *p* = 0.880) or LLRs (*r* = −0.335, *p* = 0.205). Furthermore, the relationship between SLR and LLR amplitudes did not reach statistical significance (*r*_*s*_ = 0.356, *p* = 0.176). For task-induced changes, the magnitude of SAI inhibition (%) was not associated with the facilitation of SLRs (*r* = 0.425, *p* = 0.101) or LLRs (*r* = −0.146, *p* = 0.589). Notably, the modulation of magnitudes of the two reflex components were also independent of each other (*r* = 0.253, *p* = 0.344).

## Discussion

A novel observation in our study was that, during static contraction of the FDI muscle at 10% MVC and 20 degrees of abduction, SAI inhibition (%) was significantly smaller during the position task than the force tasks. In line with previous reports [1–3], the amplitude of the heteronymous SLR was significantly greater during the position than force task. Furthermore, the amplitude of heteronomy LLR was greater during the position than force task, as reported in earlier studies [2, 3]. Importantly, comprehensive correlation analyses revealed no significant associations among SAI inhibition (%), SLR and LLR amplitudes, either within each task condition or in their task-induced changes. These findings suggest that the position task requires more proprioceptive information and involves different sensorimotor integration mechanisms compared to the constant finger force task.

In this study, we found that SAI inhibition (%) was weaker during movement (force and position tasks) compared with rest. This pattern has also been described in previous studies [25], suggesting that voluntary motor commands may partially override or modulate afferent inhibition. More importantly, SAI inhibition (%) was weaker in the position than force tasks. Before interpreting our results, it should be noted that under the same experimental setup used in our SAI experiment, the latency of the N20 component of SEP evoked by median nerve stimulation did not show significant differences between the force and position tasks [2, 20], indicating that load-related differences in stimulation timing for SAI measurement are unlikely to account for the present results.

The greater reduction of SAI observed during the position task may be related to SEP gating, as suggested by previous research [26–28]. SEP gating is the phenomenon where SEPs are reduced during voluntary movement compared to rest [29–33], which is believed to reflect the filtering of irrelevant sensory information during movement, allowing only essential inputs to be processed [30, 31, 34]. This mechanism, which modulates the influence of sensory input on the motor cortex, helps optimize motor responses and varies according to different motor perceptual demands [20, 35, 36]. In previous studies of position and force tasks, the P45 component was found to be larger during the position task, while the N33 component was larger during the force task, indicating different cortical processing of sensory inputs [2, 20]. Since the peripheral mixed nerve stimulation used in this study to evaluate SAI primarily stimulates larger diameter Group Ia sensory nerves, the position task which requires more proprioceptive information may have involved greater filtering of information from Group Ia sensory nerves derived from the electrical stimulation. This could have resulted in decreased inhibitory input from S1 to M1, consequently leading to reduced SAI.

The gating of SEPs, interpreted as being related to the attenuation of SAI, arises not only from the competition between afferent signals following electrical stimulation and those associated with the motor output itself (afferent gating) but also from a central mechanism where output from the motor center suppresses the sensory afferent pathway (efferent gating). This is supported by findings showing that SEP gating can occur before movement onset [2]. Given that SEP gating can occur at any stage along the afferent sensory pathway, or within the cerebral cortex, the mechanism underlying the reduced SAI in the position task relative to the force task should be explored in the context of efferent gating mechanisms. However, this interpretation should be made with caution, as the proposed link between SAI modulation and SEP gating is inferred from previous studies employing the same task parameters [2, 20], and SEP gating was not directly assessed in the present study.

The amplitudes of SLRs and LLRs have been reported to be greater during the position than force task [1–3], and our findings are consistent with the previous studies. Furthermore, the results of the present study, conducted at 10% MVC, were similar to those of previous studies conducted at 20% MVC [2, 3] or at multiple load intensities of 20%, 40%, and 60% MVC [1], supporting the view that differences in reflex responses depend on the load type rather than differences in voluntary contraction force [1]. SLR is known to be regulated exclusively by Ia afferents and spinal neural networks [4], and its amplitude can serve as a surrogate marker for presynaptic inhibition of Ia afferents [5]. The observed increase in SLR amplitude during the position task thus suggests that Ia afferent excitation was enhanced under this condition. Meanwhile, LLRs involve spinal circuits and supraspinal circuits including M1 [7, 37–40]. Therefore, the increased amplitude of LLRs can indicate enhanced excitability of spinal α-motoneurons projected from the M1.

The absence of significant covariation between SAI inhibition (%) and heteronymous reflex amplitudes, as well as stable BEMG, does not support a simple account in which load-type effects are governed by a single shared gain factor. Rather, the pattern is consistent with partly separable physiological mechanisms operating at different levels of the sensorimotor system. Specifically, SAI is widely interpreted as afferent-driven inhibition within sensorimotor cortical circuits (such as S1–M1 interactions), for which cholinergic involvement and GABAergic inhibitory mechanisms have been implicated [9, 10, 41]. In contrast, heteronymous SLR/LLR amplitudes primarily index the regulation of feedback gain expressed within spinal and long-loop pathways during ongoing motor control [3, 4, 42]. A more plausible interpretation, therefore, is that load type may modulate cortical afferent inhibition and reflex feedback gains in parallel, but these two processes do not necessarily exhibit a tight linear association across conditions or in task-induced changes. This interpretation is further supported by the absence of load-type differences in background EMG activity and N20 latency, making generalized background excitability or stimulus-timing differences unlikely explanations for the present pattern.

To prevent simultaneous activation of the FDI and its antagonist and to minimize contamination of the F-wave recordings [24], most previous studies have used a heteronymous pathway to elicit the SLR from the FDI [1, 3, 43]. Since the F-wave represents the response of motor neurons to an antidromic volley [44, 45], it does not occur when activating a heteronymous pathway. On the other hand, co-contraction can occur under unstable load conditions, significantly increasing the background muscle activity of the agonist group [46, 47], which may contribute to increased heteronymous SLR amplitudes. However, both in previous studies and the present one, we did not observe an increase in muscle activity during the unstable position task compared to the stable force task. Nonetheless, it remains possible that the activity of muscles involved in abduction (such as the palmar interosseous muscle) increased to an extent that did not affect the abduction torque of the index finger. Recently, a non-invasive method for measuring hand muscle activity using surface EMG has been developed [48]. Future studies should adopt this method to conduct more detailed investigations.

### Limitations and future directions

First, several methodological aspects regarding SAI measurement parameters warrant attention. The intensity of the electrical stimulation (1.2 × motor threshold) for the median nerve may have approached the saturation point of the sensory nerve action potential (SNAP). Future studies should optimize this by selecting a conditioning intensity between 25 and 40% of SNAP (max), as determined by an SNAP stimulus–response curve [16]. Additionally, the TMS intensity used to evoke 1 mV MEPs target for TS intensity might have been too strong. Higher TMS intensities can paradoxically reduce or abolish SAI, which could decrease sensitivity to inhibition [14]. A better approach is to construct an MEP input–output curve at rest [49] and select an intensity that elicits 0.5–1.0 mV MEPs. This intensity should be applied consistently across conditions to ensure a sensitive SAI measurement. Second, no data on task performance during the force and position tasks were collected. While the primary aim of this study was to compare SAI, SLR, and LLR between these two tasks, task performance data would have strengthened our discussion. Third, we did not assess fatigue during the experiment. Although there were no differences in EMG amplitude between tasks, the potential effects of fatigue should have been considered. Future studies should incorporate fatigue assessments to determine whether it influences SAI, SLR, and LLR responses. Finally, we only focused on SAI, despite the widely recognized interaction between SAI and short-interval cortical inhibition (SICI) [10, 11, 50]. In particular, it has been suggested that a decrease in SAI could be accompanied by an increase in SICI [11]. Thus, whether SICI also exhibits the load-dependent difference in SAI remains to be elucidated. Moreover, intraneural microsimulation has been considered an ideal method for investigating cutaneous versus proprioceptive contributions [11]. Future investigations should consider this method to study SAI during the force and position tasks. Additionally, future studies should utilize electroencephalography to elucidate the neural mechanisms further directly (e.g., association between task-related SAI reduction and SEP gating) underlying the difference in SAI between these two tasks. Incorporating additional sensory inputs, such as visual and auditory cues, may also provide a more comprehensive understanding of how these factors modulate sensorimotor integration and SAI during position and force tasks.

## Conclusion

This study demonstrated a significantly smaller SAI during position than force task. This finding highlights the load type-specific modulation of SAI, reflecting distinct sensorimotor processing strategies that are adapted to different load conditions.

## Supplementary Information

Supplementary Material 1. Figure S1. Raw SEP waveform from a representative subject. Red line indicates average SEP waveform recorded from C3’. The latency of the SEP component of N20 was measured to determine the individualized interstimulus interval for SAI measurement. Negativity is plotted upward

## Data Availability

The anonymized dataset generated during the present study are available in the Open Science Framework (OSF) repository at: https://osf.io/fb46s/overview?view_only=5b13910172e4464dacf96c512d11396d

## Abbreviations

SAI: Short latency afferent inhibition
MVC: Maximum voluntary contraction
M1: Primary motor cortex
S1: Primary somatosensory cortex
TMS: Transcranial magnetic stimulation
MEP: Motor evoked potential
SEP: Somatosensory evoked potential
SLRs and LLRs: Heteronymous short and long latency reflexes
FDI: First dorsal interosseous muscle
EMG: Electromyography
TS: Test stimulus
ANOVA: Analysis of variance
AER: Absolute error rate
BEMG: Background EMG
SNAP: Sensory nerve action potential
SICI: Short-interval cortical inhibition
