# Supplementary figures and images for: Short latency afferent inhibition differs with load type during isometric finger abduction
Source: J Physiol Anthropol. 2026 Feb 25;45:7. doi: 10.1186/s40101-026-00424-y (PMC13041142; doi:10.1186/s40101-026-00424-y)

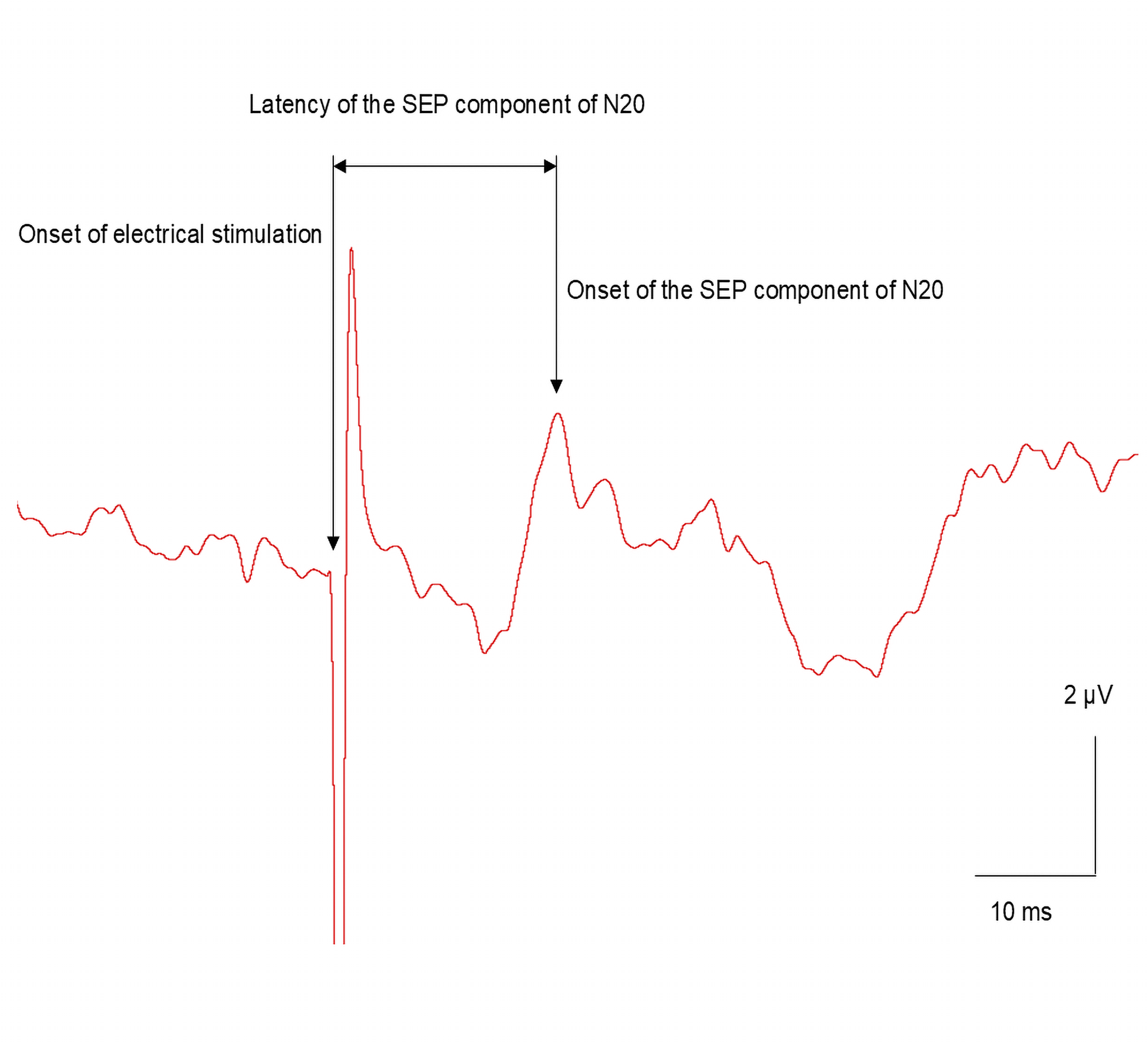

Supplement: Supplementary file 1 — Supplementary Material 1. Figure S1. Raw SEP waveform from a representative subject. Red line indicates average SEP waveform recorded from C3’. The latency of the SEP component of N20 was measured to determine the individualized interstimulus interval for SAI measurement. Negativity is plotted upward [file 40101_2026_424_MOESM1_ESM.tif]
